# Family COVID-19 cluster analysis of an infant without respiratory symptoms

**DOI:** 10.1590/0037-8682-0494-2020

**Published:** 2020-08-26

**Authors:** Myrela Conceição Santos de Jesus, Alexandra Giovanna Aragão Lima, Victor Santana Santos, Luciane Moreno Storti-Melo, Tatiana Rodrigues de Moura, Rafaela Windy Farias dos Santos, Cliomar Alves dos Santos, Paulo Ricardo Martins-Filho, Luis Eduardo Cuevas, Ricardo Queiroz Gurgel

**Affiliations:** 1 Universidade Federal Fluminense, Programa de Pós-Graduação Stricto Sensu em Microbiologia e Parasitologia Aplicadas, Niterói, RJ, Brasil.; 2 Universidade Federal de Sergipe, Programa de Pós-Graduação Stricto Sensu em Biologia Parasitária, São Cristóvão, SE, Brasil.; 3 Universidade Federal de Alagoas, Departamento de Enfermagem, Arapiraca, AL, Brasil.; 4 Universidade Federal de Sergipe, Departamento de Biologia, São Cristóvão, SE, Brasil.; 5 Universidade Federal de Sergipe, Departamento de Morfologia, São Cristóvão, SE, Brasil.; 6 Universidade Federal de Sergipe, Programa de Pós-Graduação Stricto Sensu em Ciências da Saúde, Aracaju, SE, Brasil.; 7 Laboratório Central de Saúde Pública de Sergipe, Aracaju, SE, Brasil.; 8 Universidade Federal de Sergipe, Laboratório de Patologia Investigativa, Aracaju, SE, Brasil.; 9 Liverpool School of Tropical Medicine, Department of Clinical Science, Liverpool, UK.; 10 Universidade Federal de Sergipe, Departamento de Medicina, Aracaju, SE, Brasil.

**Keywords:** Asymptomatic cases, Coronavirus, COVID-19, SARS-CoV-2

## Abstract

Diagnosing cases of coronavirus disease (COVID-19) with only non-respiratory symptoms has been challenging. We reported the diagnosis of a child who tested positive for COVID-19 with abdominal pain/diarrhea and tracked his family cluster. One member of the family tested positive for COVID-19 on real-time reverse-transcription polymerase chain reaction assay and three other family members had anti-SARS-CoV-2 antibodies.

## INTRODUCTION

Coronavirus disease (COVID-19), an emergent infectious disease caused by the severe acute respiratory syndrome coronavirus 2 (SARS-CoV-2), is a current global health emergency^1^. The confirmation of presumptive cases is often instigated by the presence of fever and respiratory symptoms^2^, although other manifestations such as gastrointestinal signs and acute diarrhea are common^3^ ^,^ ^4^. A gastrointestinal presentation, without respiratory symptoms, poses important challenges, as diarrhea is one of the main reasons for emergency consultations at pediatric clinics and the presentation of COVID-19 makes it indistinguishable from other causes of diarrhea. Moreover, although the investigations of secondary cases among contacts of confirmed COVID-19 cases is an important strategy for control, this is rarely conducted for contacts of individuals who primarily present with a complaint of diarrhea.

Here, we report the case of a child with COVID-19 who attended an outpatient clinic in Aracaju, Northeast-Brazil, with gastrointestinal symptoms and no respiratory problems and the subsequent screening of his close family members.

## CASE REPORT

### The index case

A 2-year and 9-month-old boy with diarrhea presented on 3^rd^ May 2020 at a private clinic. The parent indicated that the child had had bloody diarrhea for two days, with 6-8 stool episodes per day and no vomiting or significant fever. The child was managed as an ambulatory patient, and the frequency and characteristics of his feces normalized (one or two times per day). Two days after the onset of symptoms (5^th^ May 2020), examination revealed an afebrile child who complained of sore throat and moderate abdominal pain. A swab of the nasopharynx/oropharynx collected on 7^th^ May 2020 (two days after) yielded positive results (cycle threshold [Ct] of 36), but a fecal sample yielded negative results. Immunoglobulin Ig M and IgG antibodies assays performed 12 days after symptom onset yielded negative results. The leukocyte count on day 12 was 8300/mm^3^, with a lymphocyte count of 5644/mm^3^ (68%). The C-reactive protein (CRP) and interleukin-6 (IL-6) levels on day 12 were within normal values. Further IgG and IgM assays performed on 21^st^ and 25^th^ May 2020 yielded negative results (Table 1).

**TABLE 1:** Results of tests performed for the COVID-19-positive patient and his family members.

Participant	Degree of contact with	Respiratory	Other	RT-qPCR	IgM (AU/mL)*	IgG (AU/mL)*	Leukocytes	Lymphocytes	Neutrophil-lymphocyte	C-reactive	IL-6^¶^
	the index case	symptoms	symptoms				(reference)	(reference)	ratio	protein^¥^	
P1 (index case)	-	No	Diarrhea	36	0.0	0.0	8300/mm^3^	5644/mm^3^	0.32/mm^3^	0.9 mg/L	0.0 pg/mL
							(4000-14000)	(1520-10500)			
Mother	Daily contact	No	No	>40	4.3	0.0	9800/mm^3^	3626/mm^3^	1.46/mm^3^	16.0 mg/L	4.9 pg/mL
							(3600-11000)	(740-5500)			
Father	Daily contact	No	No	>40	0.5	16.4	7700/mm^3^	3626/mm^3^	0,82/mm^3^	1.5 mg/L	7.3 pg/mL
							(3600-11000)	(740-5500)			
Sister	Daily contact	No	No	>40	0.1	1.8	4500/mm^3^	1935/mm^3^	1.02/mm^3^	4.9 mg/L	3.0 pg/mL
							(3600-11000)	(740-5500)			
Aunt	3 times per week - 2	No	No	>40	0.6	0.0	6600/mm^3^	2310/mm^3^	1.40/mm^3^	4.1 mg/L	0.0 pg/mL
	hours each time						(3600-11000)	(740-5500)			
Uncle	3 times per week - 2	No	No	37	0.3	0.0	5600/mm^3^	2072/mm^3^	1.21/mm^3^	1.9 mg/L	3.3 pg/mL
	hours each time						(3600-11000)	(740-5500)			
Cousin	3 times per week - 2	No	Diarrhea	>40	0.6	0.0	7200/mm^3^	2592/mm^3^	1.53/mm^3^	5.9 mg/L	5.9 pg/mL
	hours each time						(3600-11000)	(740-5500)			

*Negative: <0.9; **Undetermined:** 0.9 to 1.1; **Positive** >1.1. ^¥^ **Reference**: 0.0-6.0 mg/L; ^¶^ **Reference**: 0.0-7.0 pg/mL.

### Investigation of contacts

The mother was a 41-year-old healthy woman who had been in isolation since 17^th^ March 2020. A nasopharyngeal/oropharyngeal swab taken on 7^th^ May 2020 yielded negative results on real-time reverse-transcription polymerase chain reaction (RT-qPCR) assay, but her IgG test performed on 15^th^ May 2020 yielded positive results while her IgM yielded negative results. Her leukocyte count and IL-6 level were normal, and her CRP level was 16.0 mg/L (Table 1). 

The father was 56 years old, asymptomatic, and employed in the oil drilling industry. He had regular contact with people outside the household until 20^th^ April 2020, his last working day, and sporadic contacts afterward when purchasing essential supplies at the local shop. He indicated he wore home-made masks and used alcohol-based hand sanitizers when shopping. A nasopharyngeal/oropharyngeal swab taken on 7^th^ May 2020 yielded negative results. IgG and IgM assays were performed on 15^th^ May 2020 and yielded values of 0.5 AU/mL (negative) and 16.4 AU/mL (positive), respectively. His leukocyte count and CRP level were normal, but his IL-6 level was just above the normal range (Table 1). He did not develop symptoms on follow up.

A 16-year-old asymptomatic sister residing in the same household had no contacts outside her home since 20^th^ April 2020 when she had visited her grandmother and relatives. Her nasopharyngeal/oropharyngeal swab (11^th^ May 2020) and IgG (15^th^ May 2020) tests yielded negative results, but her IgM (15^th^ May 2020) assay yielded positive results. Her leukocyte count, CRP level, and IL-6 level were normal (Table 1). 

Another 41-year-old asymptomatic aunt who had visited the household on 28^th^ April 2020 had not been in contact with the child since his symptoms started. Her RT-qPCR (15^th^ May 2020), IgG, IgM, leukocyte count, CRP, and IL-6 tests yielded normal results (Table 1).

A 45-year-old asymptomatic uncle, who was unable to maintain social isolation due to work commitments had a positive nasopharyngeal/oropharyngeal RT-PCR assay result on 15th May 2020, but his IgM and IgG tests performed on the same day yielded negative results. His leukocyte count, CRP level, and IL-6 level were normal (Table 1).

Lastly, a 19-year-old female cousin who had maintained social isolation since 17^th^ March 2020 and with contact with her parents and relatives studied here. On 9^th^ and 10^th^ May 2020, she had presented at an emergency department on account of a 2-day history of abdominal pain and diarrhea. On 15^th^ May 2020, her nasopharyngeal/oropharyngeal swab yielded negative results, and her IgM and IgG samples collected 21 days after symptom onset yielded negative results. Her leukocyte count, CRP level, and IL-6 level were normal (Table 1). All relatives were followed until 26^th^ May 2020, 23 days after the onset of the child’s symptoms.

This is a case report comprising a child with a chief complaint of diarrhea and the clinical history of his six contact family members during the 20 days prior to the onset of his symptoms. 

The index patient’s parents, and sister lived with him in the same house and, therefore, had daily contact. His uncle, aunt, and cousin had contact with him three times per week, for two hours at each meeting. Contact with the uncle and cousin was disrupted when the child’s symptoms began, and the diagnosis was made (Table 1).

Demographic information, medical history, clinical information, and laboratory findings were collected following standard protocols. Laboratory parameters included blood tests, CRP, IgM and IgG antibodies, and leukocyte profile.

RNA for RT-qPCR assays were extracted from nasopharyngeal/oropharyngeal swabs and stool samples using the BioGene DNA/RNA Extractor Kit (Bioclin, Minas Gerais, Brazil). The RT-PCR assay was conducted using the BIOMOL OneStep/COVID-19 (BioManguinhos, Oswaldo Cruz Foundation, Brazil), on an Applied Biosystems 7500 thermocycler. 

Immunoassays for serum IgM and IgG antibodies against SARS-CoV-2 were performed using a qualitative fluorescence immunoassay (Ichroma^TM^ COVID-19, Boditech Med Inc, Republic of Korea). 

The serum IL-6 concentration was measured using an enzyme-linked immunosorbent assay (ELISA) (eBioscience, San Diego CA, USA). A standard curve was generated for each set of samples following the manufacturer’s instructions.

## DISCUSSION

The diagnosis of COVID-19 without fever or respiratory symptoms requires a high degree of clinical suspicion or epidemiological links. We report here the case of a child with COVID-19 whose main presentation comprised gastrointestinal symptoms without respiratory symptoms. We also describe the clinical findings, molecular and serological assay results of the family members with whom he had been in contact up to 20 days before symptom onset. Although all contacts were asymptomatic, the molecular and serological assays demonstrated that several relatives had current or past SARS-CoV-2 infections.

Children with COVID-19 are more likely to be asymptomatic or have mild symptoms than adults^5^. Half of children may have gastrointestinal symptoms in the absence of respiratory symptoms^4^. Angiotensin-converting enzyme 2 (ACE2) receptors are highly expressed in the small intestine, which may lead to intestinal inflammation and diarrhea^6^. In the present case, SARS-CoV-2 RNA was detected in samples from the respiratory tract, although the main presentation was a gastrointestinal complaint.

RNA SARS-CoV-2 can be detected in fecal samples and its shedding may be present in feces for a longer time than that in the respiratory tract^7^. However, the stool sample of the index patient yielded negative results, but the two respiratory samples yielded positive results. The child’s source of infection was unknown but could have originated from any of the three family members with SARS-CoV-2 antibodies. The child’s father and sister had positive IgG test results, while the mother had positive IgM test results. One family member had a positive RT-qPCR assay result. As five of the six relatives were asymptomatic, it is possible that asymptomatic transmission had played a role in transmission, which is considered to represent and attributable fraction of 50-80% of incident infections^8^. 

Our index patient did not have detectable antibodies even at 23 days after symptom onset. Non-seroconversion has been reported for SARS-CoV-2, SARS-CoV, and Middle East respiratory syndrome^9^.

In summary, the present study showed the importance of clinical manifestations other than respiratory for the suspicion and diagnosis of COVID-19 among children with mild gastrointestinal symptoms. We demonstrated how contact tracing can document the spread of infection within a family nucleus. Based on the identification of positive cases of COVID-19, it is necessary to either investigate potentially transmitting contacts or indicate their self-isolation.

## References

[1] World Health Organization (WHO) (2020). IHR Emergency Committee on Novel Coronavirus (2019-nCoV).

[2] Chavez S, Long B, Koyfman A, Liang SY (2020). Coronavirus Disease (COVID-19): A primer for emergency physicians. Am J Emerg Med.

[3] Huang C, Wang Y, Li X, Ren L, Zhao J, Hu Y (2020). Clinical features of patients infected with 2019 novel coronavirus in Wuhan, China. Lancet.

[4] Tian Y, Rong L, Nian W, He Y (2020). Review article: gastrointestinal features in COVID-19 and the possibility of faecal transmission. Aliment. Pharmacol. Ther.

[5] Lu X, Zhang L, Du H, Zhang J, Li YY, Qu J (2020). SARS-CoV-2 Infection in Children. N Engl J Med.

[6] Liang W, Feng Z, Rao S, Xiao C, Xue X, Lin Z (2020). Diarrhoea may be underestimated: a missing link in 2019 novel coronavirus. Gut.

[7] Santos VS, Gurgel RQ, Cuevas LE, Martins-Filho PR (2020). Prolonged fecal shedding of SARS-CoV-2 in pediatric patients. A quantitative evidence synthesis. J Pediatr Gastroenterol Nutr.

[8] Li R, Pei S, Chen B, Song Y, Zhang T, Yang W (2020). Substantial undocumented infection facilitates the rapid dissemination of novel coronavirus (SARS-CoV2). Science.

[9] Dijkman R, Jebbink MF, El Idrissi NB, Pyrc K, Müller MA, Kuijpers TW (2008). Human coronavirus NL63 and 229E seroconversion in children. J. Clin. Microbiol.

